# Occupational Post-Exposure Prophylaxis (PEP) against Human Immunodeficiency Virus (HIV) Infection in a Health District in Cameroon: Assessment of the Knowledge and Practices of Nurses

**DOI:** 10.1371/journal.pone.0124416

**Published:** 2015-04-16

**Authors:** Leopold Ndemnge Aminde, Noah Fongwen Takah, Anastase Dzudie, Neville Mengnjo Bonko, George Awungafac, Divine Teno, Lawrence Mbuagbaw, Karen Sliwa

**Affiliations:** 1 Department of Medicine, Faculty of Health Sciences, University of Buea, Buea, Cameroon; 2 Global Health Systems Solutions, Limbe, Cameroon; 3 Department of Medicine, Faculty of Health Sciences, University of Buea, Buea, Cameroon and Department of Internal Medicine, General Hospital Douala, Douala, Cameroon and Department of Medicine, Faculty of Health Sciences, University of Cape Town, Cape Town, South Africa; 4 Department of Medicine, Faculty of Health Sciences, University of Buea and Baptist Hospital, Mutengene, Cameroon; 5 Tubah District Hospital, Bambui, Cameroon; 6 Department of Clinical Epidemiology & Biostatistics, McMaster University, Hamilton, ON, Canada; 7 Hatter Institute of Cardiovascular Research in Africa, Department of Medicine, Faculty of Health Sciences, University of Cape Town, Cape Town, South Africa; Alberta Provincial Laboratory for Public Health/ University of Alberta, CANADA

## Abstract

**Background:**

Health care providers are at risk of acquiring human immunodeficiency virus (HIV) infection from occupational exposure, with nurses being the most vulnerable. There is no data on the awareness of post-exposure prophylaxis (PEP) among nurses in Cameroon. This study aimed to assess the knowledge, practices of nurses regarding PEP for HIV and their determinants in Cameroon.

**Methods:**

A cross-sectional study was conducted between April and July 2013, and involved 80 nurses in a rural health district in the North West Region of Cameroon. Data was collected using a structured questionnaire and analysed using the SPSS software version 20.

**Results:**

In all, 73.7% of the participants had poor knowledge about PEP for HIV. Though many (83.8%) had heard about PEP, just 10 (12.5%) had received formal training on PEP for HIV. Only 24 (30%) and 20 (25%) knew the correct drug regimen and duration of treatment respectively. The majority (85%) considered themselves to be at risk of acquiring HIV at work, with 54 (67.5%) having experienced an exposure in the past, mainly while setting up intravenous lines (57.4%), recapping needles (37.0%) and during delivery (24.1%). Of those exposed, ten (18.9%) received PEP, which was started after 24 hours in 50%. In multivariable regression analyses, awareness of hospital policy [OR: 0.043 (0.005–0.404), p-value = 0.006] was associated with Good knowledge on PEP for HIV.

**Conclusions:**

The knowledge and practice of nurses on PEP for HIV in Cameroon is low. There is urgent need for training programmes and workshops to increase awareness, improve practice, and reduce the risk of HIV acquisition from work related activities among health care providers.

## Introduction

Human Immunodeficiency virus (HIV) infection and Acquired Immunodeficiency Syndrome (AIDS) are established public health problems, particularly in sub-Saharan Africa where they affect even health workers [1]. The advent of Antiretroviral Therapy (ART) has significantly improved the management and prevention of HIV infection including those at risk through programmes such as the PMTCT (Prevention of Mother to Child Transmission) [2], PEP (Post exposure prophylaxis) [3] and more recently PrEP (Pre exposure prophylaxis) [3,4].

PEP consists of administering a short course of ART to reduce the likelihood of seroconversion following events with high risk of exposure to HIV [5]. The overall PEP process involves first aid, counselling, risk assessment, relevant laboratory investigations with the consent of the exposed individual and source, followed by provision of a short course of ART for a period of 28 days, and monitoring [5,6]. PEP is said to prevent about 81% of seroconversion [7] and is at present the only means of reducing the risk of HIV infection after exposure. Practices known to be unsafe such as re-use of inadequately sterilised needles, careless handling of contaminated needles, poor hazardous waste management, have the potential to increase the risk of acquiring blood borne pathogens [8]. The World Health Organisation (WHO) reports that, about 3 million percutaneous occupational exposures to blood or other bodily fluids occur in health care settings; the majority (90%) of which occurred in developing countries [7]. The average risk of HIV acquisition after percutaneous exposure to infected blood is estimated to be 0.3% and about 0.09% after exposure to mucous membrane [9]. The risk of acquiring blood borne pathogens is high in Africa, most probably reflecting the high prevalence of those conditions in the African setting.

Some studies have reported favourable knowledge on PEP among healthcare workers [6,10], but several others have rather found important knowledge gaps on PEP among healthcare workers. In Nepal, only 6% of Nurses in Chitwan Medical College Teaching Hospital had good knowledge on PEP [11], while in Zimbabwe 65% of healthcare workers [12] and 83% in Ethiopia had poor knowledge on PEP. Furthermore, among the exposed respondents, 81.6% did not use PEP with 33.8% of them reporting lack of knowledge on the use of PEP[1]. Similarly, inadequate knowledge on PEP has been reported among medical doctors in a tertiary hospital in Nigeria [13]. With evidence from studies suggesting that nurses are at higher risk of occupational acquisition of HIV through needle stick injuries and contact with infected body fluids [11,14], it is important for nurses to have adequate knowledge on how to protect themselves.

Currently, there is no study on PEP for HIV in Cameroon. This study was thus conducted to assess the knowledge and practices of nurses, as well as factors associated with good knowledge in a rural Health District in Cameroon, on PEP for occupational acquisition of HIV infection.

## Materials and Methods

### Study design and Area

This cross-sectional study was conducted from April 1 to July 15, 2013 among nurses in Tubah Health District in the North West Region of Cameroon. Tubah Health District is a rural area with a population of 53,988 inhabitants and it has a District Hospital and 10 satellite Integrated Health Centres.

### Sample Size and Sampling Technique

A convenience sampling technique was employed. Of the 85 eligible nurses working in the Health District, 80 nurses were included.

### Data Collection and Study Procedure

A structured self-administered English language questionnaire was prepared by the research team based on published studies [6,9,11,15]. It contained socio-demographic characteristics and questions to assess knowledge and practice regarding PEP for HIV. The validity of the contents of the developed questionnaire was established through consultation with experts. The questionnaire was pretested in a sample comprising 10% of the 95 nurses working in the health district. Ten nurses were thus involved in the pre-test and were eventually restricted from participating in the main study. Hence, 85 nurses were eligible to take part in the main survey. The questionnaire contained questions on socio-demographic characteristics, 11 questions on knowledge and 11 questions on practices. Questions assessing knowledge included if participant had ever heard about PEP; the sources of knowledge; if they had ever had a training on PEP; if they were aware of the hospital policy on PEP for HIV; what to do in case of exposure, indications, drugs and drug regimens for PEP for HIV. Practice questions included what participants did in case of exposure, if sources of exposure and the exposed were screened for HIV with reasons for not doing so where applicable, if they took PEP (for those exposed) and time lapse from exposure to starting PEP.

### Scoring of Knowledge of Participants

Each of the 11 questions on knowledge was equitably scored and respondents who had greater than or equal to 8 correct responses (≥70%) were considered to have “Good knowledge”, those with 6 to 7 (50–69%) correct responses were considered as having “Average knowledge” while those with less than or equal to 5 correct responses (< 50%) were considered to have “Poor knowledge”. For purposes of analysis to determine factors associated with good knowledge, the population was divided into two groups: participants with Poor knowledge and those with Average-to-Good knowledge.

Regarding participants’ practices, there were twelve questions which assessed circumstances of exposure and practice of nurses. The practices were simply evaluated based on correct responses on practices stipulated by guidelines at the time.

### Data Analysis

Data was entered, cleaned and then analysed using Statistical Package for Social Sciences (SPSS) IBM v. 20. The data was summarised as frequencies, percentages, means and standard deviations, where applicable. Factors associated with good knowledge were investigated using logistic regressions. A p-value ≤ 0.05 was considered statistically significant.

### Ethical Consideration

Approval was obtained from the Regional Delegation of the Ministry of Public Health (MOH) and authorities of the District Health Service, District Hospital and Health Centres. Nurses were included in the study after they had been explained the aims of the study and was followed by their written and oral consent to the study. Confidentiality of the study participants was also maintained.

## Results

### Sociodemographic Characteristics (Table 1)

In all, 80 properly completed questionnaires were returned from the 85 eligible nurses in the health district, giving a response rate of 94.1%. Most (66.3%) of the participants were females. The mean age was 34±8 years (range: 21–55 years), with majority (43.8%) of participants aged between 20 to 30 years. Seventy-one (88.8%) of them were Christians and most (62.5%) had attained secondary education. Forty-three (53.7%) of the nurses were single. Furthermore, the majority (47.5%) of the nurses had served for a period 1–5 years in hospital with 65% of them being in medical units (General Medicine and Paediatric Wards) and 35% in surgical units (Surgery and Maternity wards).

**Table 1:** Socio-Demographic Characteristics of Nurses in Tubah Health District, 2013.

Variables		N (%)
Age	20–30 years	35 (43.8)
	31–40 years	28 (35.0)
	41–50 years	14 (17.4)
	>50 years	3 (3.8)
Gender	Male	27 (33.7)
	Female	53 (66.3)
Religion	Christian	71 (88.8)
	Muslim	9 (11.2)
	Other	0 (00.0)
Educational level	Primary	9 (11.3)
	Secondary	50 (62.5)
	University	21 (26.2)
Length of Service	< 6months	4 (5.0)
	6–12 months	10 (12.5)
	1–5 years	38 (47.5)
	5–10 years	9 (11.3)
	>10years	19 (23.7)
Marital Status	Single	43 (53.7)
	Married	35 (43.7)
	Divorced	2 (2.6)
Unit of Work	Medical (Medicine & Paediatrics)	52 (65.0)
	Surgical (Maternity & Surgery)	28 (35.0)

### Knowledge of Nurses about PEP for HIV (Tables 2 and 3)

Over two-thirds (73.7%) of the participants in the study had poor knowledge about PEP for HIV.

**Table 2:** Knowledge about PEP for HIV among Nurses in Tubah Health District, 2013.

Variables	Responses	Frequency
Heard about PEP	Yes	67 (83.8)
	No	13 (16.2)
Source of Knowledge of PEP (multiple responses)	Newspaper/Journal	03 (4.5)
	Radio	06 (8.9)
	Television	01 (1.5)
	Seminar/ Workshop	24 (35.8)
	Ward Rounds	30 (44.8)
	PEP training	04 (5.9)
	Can’t remember	09 (13.4)
Aware of Hospital PEP policy	Yes	11 (13.9)
	No	69 (86.1)
Ever had training on PEP?	Yes	10 (12.5)
	No	70 (87.5)
What proportion of needle prick injuries result in HIV transmission?	1/100	20 (25.0)
	1/500	21 (26.3)
	3/1000	08 (10.0)
	10/1000	19 (23.7)
	Don’t know	12 (15.0)
Which of the following are high risk fluids for transmission of HIV? (multiple answers)	Breast milk	65 (81.3)
	Urine	02 (2.5)
	Peritoneal fluid	13 (16.3)
	Saliva	29 (36.3)
	Pleural fluid	17 (21.3)
	Cerebro-spinal fluid	16 (20.0)
	Faeces	01 (1.3)
	Synovial fluid	15 (18.7)
Indication for initiation of PEP (multiple answers acceptable)	Needle prick injury	63 (79.7)
	Splashing of blood/bodily fluid on mucosal surfaces	19 (24.1)
	Rape	41 (51.9)
	Infants born HIV positive mothers	41 (51.9)
First aid measure to institute following needle stick injury	Promote active bleeding of the wound	41 (51.3)
	Wash thoroughly with soap and water	38 (47.5)
	Don’t know	01 (1.2)
How soon after needle prick should PEP be started?	Within 1 hour	53 (66.3)
	After 72 hours	22 (27.5)
	Don’t know	05 (6.2)
What is the ideal HIV-PEP regimen following needle stick injury?	One drug regimen	13 (16.3)
	Two drug regimen	36 (45.0)
	Expanded three drug regimen	24 (30.0)
	Don’t know	07 (8.7)
Which of the following drugs are used in PEP (multiple answers acceptable)	Zidovudine	46 (57.5)
	Glymepiride	06 (7.5)
	Jevirapine	10 (12.5)
	Lamivudine	38 (47.5)
	Levamisole	08 (10.0)
	Stavudine	20 (25.0)
	Famotidine	04 (5.0)
	Nevirapine	51 (63.8)
Duration of PEP	For life	09 (11.3)
	4 weeks	20 (25.0)
	28 weeks	19 (23.7)
	6 months	03 (3.7)
	2 weeks	09 (11.3)
	Don’t know	20 (25.0)

**Table 3:** Level of Knowledge and mean score of Nurses on PEP for HIV, Tubah, 2013

Level	N (%)	Mean±SD
Good (>75%)	05 (6.3)	8.5±0.5
Average (50–75%)	16 (20.0)	6.0±0.5
Poor (<50%)	59 (73.7)	3.5±1.5

A good proportion (83.8%) of the participants had heard about post-exposure prophylaxis for HIV. The main source of information about PEP was from ward rounds (44.8%) with only an eighth (12.5%) of the nurses admitting to have had a formal training on post-exposure prophylaxis for HIV. Only eleven (13.9%) participants were aware of their hospital PEP policies. In addition, only eight (10%) nurses knew the proportion of needle pricks that result in HIV transmission.

Regarding participants’ knowledge on high risk fluids for HIV transmission, their responses were quite poor, as less than a fifth could correctly identify potentially high risk fluids; peritoneal fluid (16.3%), synovial fluid (18.7%), pleural fluid (21.3%), cerebro-spinal fluid (20.0%) respectively, excluding breast milk (81.3%) which was correctly identified. In all, sixty-three (79.7%), forty-one (51.9%), forty-one (51.9%), of the participants knew that needle prick, rape and infants born to HIV positive mothers, respectively, were indications for initiation of post-exposure prophylaxis, though only 24.1% knew that splashing of an infected individual’s blood or bodily fluid on mucosal surfaces was also an indication for PEP.

Almost all (98.8%) of the participants correctly identified the appropriate first aid measure to institute in case of needle stick injury. Fifty-three (66%) of the nurses knew how soon PEP was to be initiated after needle prick.

With respect to the ideal PEP drug regimen, only twenty-four (30.0%) participants correctly stated the expanded 3 drug regimen, while thirty-six (45.0%) and thirteen (16.3%) incorrectly stated two-drug and one-drug regimens respectively and seven (8.7%) did not know at all. Overall, fifty-one (63.8%) identified Nevirapine, forty-six (57.5%) identified Zidovudine, thirty-eight (47.5%) identified Lamivudine and twenty (25%) identified Stavudine correctly as drugs that may be used in PEP. Six (7.5%), ten (12.5%), eight (10.0%) and four (5.0%) participants incorrectly mentioned Glymepiride (sulphonylurea—anti-diabetic drug), Jevirapine (non-existing drug), Levamisole (anti-parasitic drug) and Famotidine (H_2_ inhibitor—drug against Peptic ulcer disease) respectively as drugs which may be used in PEP.

Only a quarter (25.0%) of the nurses knew the correct duration of therapy. Nine (11.3%), nine-teen (23.7%), three (3.7%) and nine (11.3%) participants incorrectly mentioned duration of PEP as; for life, 28 weeks, 6 months and 2 weeks, respectively.

### Practices status of Nurses towards PEP for HIV (Table 4)

Sixty-eight (85.0%) nurses in the study considered themselves at risk of acquiring HIV and fifty-four (67.5%) admitted to have had occupational exposure to HIV. Majority (63.0%) of the exposures were needle pricks while thirteen (24.0%) had both needle prick and splashing of blood/bodily fluid on mucosal surfaces.

**Table 4:** Risks, Exposures and Practices of PEP for HIV among Nurses in Tubah Health District, 2013.

Questions	Responses	N (%)
Do you consider yourself to be at risk of HIV acquisition at your work place?	Yes	68 (85.0)
	No	12 (15.0)
Have you ever had occupational exposure to HIV in the past?	Yes	54 (67.5)
	No	26 (32.5)
What type of exposure was it? (N = 54)	Needle prick	34 (63.0)
	Splashing of blood/bodily fluid on mucosal surfaces	07 (13.0)
	Both Needle prick and splashing of blood on mucosal surface	13 (24.0)
How many exposures have you had in 12months? (N = 54)	1	29 (53.7)
	2–3	17 (31.5)
	>4	08 (14.8)
What were circumstances of exposure? (multiple answers accepted)	Setting up IV line	31 (57.4)
	During surgery	03 (5.6)
	Giving injections	12 (22.2)
	Collecting blood samples	05 (9.3)
	Recapping needles	20 (37.0)
	During delivery	13 (24.1)
	Other	01 (1.9)
If you have had occupational exposure to HIV, did you screen or test for HIV? (N = 54)	Yes	39 (72.2)
	No	15 (27.8)
If No, why did you not test for HIV? (N = 15)	Not aware	02 (13.3)
	Assumed patient was HIV negative	08 (53.3)
	Other reasons	05 (33.3)
Did you receive PEP after exposure? (N = 54)	Received	10 (18.9)
	Did not receive	44 (81.1)
What was the time lapse from exposure to which PEP was received after exposure? (N = 10)	< 24hours	05 (50.0)
	> 24hours	05 (50.0)
Reason for not receiving PEP? (N = 44)	Not necessary	03 (6.8)
	ARVs not available	01 (2.3)
	Source HIV was negative	21 (47.7)
	Not aware of need to take PEP after exposure	04 (9.1)
	Not aware of hospital protocol concerning PEP at the time	07 (15.9)
	Did not believe I could be HIV positive	08 (18.2)
Post exposure screening of the source exposure? (N = 54)	Screened	30 (55.6)
	Not screened	24 (44.4)
What was the HIV status of the exposure? (N = 30)	Positive	08 (26.7)
	Negative	22 (73.3)

Within the last 12 months, twenty-nine (53.7%), seven-teen (31.5%) and eight (14.8%) respondents had had 1, 2 to 3 and greater than 4 exposures respectively. The main circumstances of exposure included; setting up intra-venous (IV) lines (57.4%), recapping needles (37.0%), during delivery (24.1%) and giving injections (22.2%).

Of the fifty-four participants who had exposures, only thirty-nine (72.2%) got tested or screened for HIV. Amongst those who did not screen for HIV, most (53.3%) assumed the source was HIV negative as their reason for not screening. 13.3% said their reason for not screening was, that they were not aware of need to screen for HIV.

Of the 54 exposed individuals, only 10 (18.9%) received PEP of which 50.0% received their PEP after 24 hours of exposure. For exposed participants who did not receive PEP, some of the reasons included; source HIV status was negative (47.7%), “did not believe I could be HIV positive” (18.2%), not aware of hospital protocol for PEP at the time (15.9%), not aware of need to take PEPs after exposure (9.1%) and three (6.8%) said it was not necessary to receive PEP after exposure as their reason for not doing so. Only thirty (55.6%) of the sources of exposures were screened for HIV, eight of whom were positive (26.7%).

### Factors associated with Knowledge on PEP for HIV among Nurses (Table 5)

Among the variables tested for association with Average to Good knowledge for PEP; nurses who had attained university education were about two times likely to have good knowledge than those who hadn’t, and nurses whose source of knowledge on PEP was from ward rounds were about three times more likely to have good knowledge than others but these associations were not statistically significant, OR: 1.6 (0.5–4.7), p-value = 0.3 and OR: 2.7 (0.8–8.2), p-value = 0.07 respectively. Nonetheless, following bivariate analysis, the following were significantly associated with good knowledge; source of knowledge on PEP from seminar/workshop [OR: 0.19 (0.06–0.58), p-value = 0.004], having had formal training on PEP [OR: 0.10 (0.02–0.46), p-value = 0.003] and awareness of hospital policy on PEP [OR: 0.01 (0.002–0.166), p-value = 0.000] which was strongly significant.

**Table 5:** Factors associated to Good Knowledge in PEP for HIV among Nurses in Tubah Health District, 2013.

Variable	Bivariate Analysis		Multivariate Analysis	
	Odd ratio (95%CI)	p-value	Odd ratio (95%CI)	p-value
University Education	1.61 (0.54–4.77)	0.393		
Length of Service > 1year	0.87 (0.24–3.13)	0.828		
Source of knowledge on PEP from Ward rounds	2.72 (0.89–8.27)	0.076	1.23 (0.298–5.155)	0.76
Had previous formal training on PEP	0.11 (0.03–0.47)	0.003*	0.36 (0.054–2.516)	0.307
Aware of hospital policy concerning PEP for HIV	0.02 (0.002–0.166)	0.000*	0.04 (0.005–0.404)	0.006^#^
Source of knowledge from Seminar/Workshop	0.19 (0.06–0.58)	0.004*	0.51 (0.126–2.095)	0.35

* = significant on bivariate analysis.

^#^ = significant in multivariate analysis.

In multivariable analysis, prior awareness of hospital policy for PEP was found to be closely associated with Good knowledge for PEP [OR: 0.043 (0.005–0.404), p-value = 0.006].

## Discussion

Adherence to the universal precaution guidelines is fundamental in the prevention of accidental acquisition of HIV infection at workplaces. Furthermore, the appropriate management of exposed individuals plays a crucial role in control and prevention of seroconversion. The paucity of published data on post exposure prophylaxis for HIV among healthcare providers in Cameroon thus motivated this study aiming to assess the knowledge and practices of nurses regarding post exposure prophylaxis for HIV.

### Knowledge of Nurses

We found that overall; nurses had a poor knowledge on PEP for HIV with over two-third of participants having a poor knowledge score. This is at variance of findings from studies among nurses Nepal and health care workers in Ethiopia, where majority had fair knowledge (68%) and adequate knowledge (63.1%) respectively on PEP for HIV[11,6]. However, our findings are similar to those of Monera and colleagues in Zimbabwe who found that 65% of their participants had poor knowledge [12].

Four in five participants had heard about PEP for HIV, essentially via participation at ward rounds. Our findings are higher than the 67.1% recorded among nursing and midwifery students in Hawassa University, Ethiopia [16]. However this is lower than the 92.8% reported in Gondar Ethiopia [6] among health care workers, the 97% reported in a tertiary care centre in Nigeria [15] and 97.7% reported among family physicians in Nigeria [17].

The main source of knowledge for participants in our study was the ward rounds. This is contrary to the findings of Jharna and colleagues among Nurses in Nepal [11] who’s knowledge on PEP was essentially from “self-learning”. Owolabi and colleagues in Nigeria [15] also had different findings, with 73.8% of their participants gaining their knowledge on PEP from seminars and workshops. In our study, only 12.5% of the participants had received formal training on PEP for HIV. The poor knowledge observed in our study is most likely due to the informal sources of information among our study participants.

Less than a fifth of our study participants could correctly identify high risk fluids for HIV transmission. This is lower than the 65% correct identification of high risk fluids among nurses in Nepal and Foster et al in which majority of their participants correctly identified non-blood high risk fluids [11,18]. Similarly, high rates of correct identification of body fluids were identified among family physicians in Nigeria [17] though studies among anaesthetists in UK and surgical residents in Nigeria also reported lower rates [19,20]. Regarding appropriate time for initiation of PEP, 66% of the nurses knew how soon PEP was to be initiated following needle stick injury. Our findings correlate with a study in Mumbai in which 64% of the participants correctly stated when to start PEP [21] and also the 60% recorded among Nurses in Nepal [11]. This is higher than the 50.8% recorded among health care workers in Gondar, Ethiopia [6]. Our findings are also higher than the 22.3% reported in a study from Mulago hospital in Uganda [22]. In another study among medical interns only 31.6% of respondents stated the correct time for initiation of PEP [23]. The differences observed in the knowledge patterns here could be attributed to the differences in overall knowledge performance of the study participants as well as differences in the study populations and health care settings.

### Practice of Nurses

The majority of the nurses (85%) considered themselves to be at risk of acquiring HIV at their work place with 54 (67.5%) admitting to have experienced such exposure in the past. This was quite comparable to the 74.5% exposure reported in a study in conducted in three tertiary hospitals in India [24]. Mathewos and colleagues in a study in Gondar found that 33.8% of their participants declared a previous occupational exposure [6] while another study in the Abuja Teaching Hospital in Nigeria found that 30.9% of their participants had past exposure. Much lower rates of exposures have been reported in Italy [25].

The circumstances of exposure included setting up intra-venous lines, recapping needles which are consistent with findings of Owolabi and colleagues and Gupta et al in India [7,15]. Despite the high rate of occupation exposure among our participants, only 10 (18.9%) received PEP with 50% receiving such treatment after 24 hours. This was slightly higher than the 6% of exposed participants who received post-exposure prophylaxis at Abuja Teaching hospital [15]. Most of our participants who didn’t receive PEP did so because the sources of exposure were HIV negative. It should however be noted that, some participants did not believe they could be infected by HIV, hence no need for PEP. The low uptake of PEP in our study is similar to reports from Uganda and Kenya [22,26].

### Factors associated with Nurses’ Knowledge for PEP

We found that source of knowledge on PEP from seminar/workshop, previous formal training on PEP for HIV and awareness of hospital policy were significantly associated with good knowledge of PEP. Agaba and colleagues found among Family Physicians that being a junior doctor and male gender was significantly associated with adequate knowledge for PEP [17]. On multivariate analysis, they however found no variable independently predictive of adequate knowledge. On the other hand in our study, awareness of hospital policy on PEP for HIV was the only independent predictor of good knowledge on multivariate analysis.

We acknowledge the cross-sectional design to be a limitation of our study. This study however is the first of its nature to be carried out in Cameroon and public health relevance of our findings cannot be overemphasised. Larger studies should be conducted involving the diversity of health care workers in the country to confirm and refine our findings.

## Conclusions

The knowledge and practices of nurses in rural Cameroon regarding post exposure prophylaxis for HIV is poor. Hospital PEP policies for HIV should be provided to all health care workers. Health education campaigns by public health authorities are paramount to improve awareness and uptake of PEP for HIV and thus reducing the risk of occupational acquisition of HIV.
